# Expectations Regarding Gastein Healing Gallery Treatment and Their Connection to Health-Related Quality of Life

**DOI:** 10.3390/ijerph20075426

**Published:** 2023-04-06

**Authors:** Loren Toussaint, Kien Huynh, Niko Kohls, Fuschia Sirois, Hannah Alberts, Jameson Hirsch, Christian Hanshans, Quang Anh Nguyen, Antje van der Zee-Neuen, Martin Offenbaecher

**Affiliations:** 1 Department of Psychology, Luther College, Decorah, IA 52101, USA; 2 Department of Neurosurgery, University of Iowa, Iowa City, IA 52242, USA; 3 Department of Social Work & Health, Coburg University of Applied Sciences and Arts, 96450 Coburg, Germany; 4 Department of Psychology, Durham University, Durham DH1 3LE, UK; 5 School of Graduate Psychology, Pacific University, Forest Grove, OR 97116, USA; 6 Department of Psychology, East Tennessee State University, Johnson City, TN 37614, USA; 7 Department of Applied Sciences and Mechatronics, University of Applied Science Munich, 80335 München, Germany; 8 Department of Psychology and Psychiatry, Mayo Clinic, Rochester, MN 55905, USA; 9 Institute of Physiology and Pathophysiology, Gastein Research Institute, Paracelsus Medical University, 5020 Salzburg, Austria; 10 Gastein Healing Gallery, 5630 Bad Hofgastein, Austria

**Keywords:** ecomedicine, Alpine health resources, speleotherapy, health expectations, health-related quality of life, Bad Gastein, Gastein Healing Gallery

## Abstract

The present study examines connections between patient expectations and health-related quality of life. We explore a key distinction between expectations about general health and expectations for functional improvement. Patients were 1444 individuals with multiple conditions experiencing chronic pain who were seeking treatment at the Gastein Healing Gallery in Böckstein, near Bad Gastein, Austria. In addition to measures of expectations, patients completed measures of pain, mental and physical health, life satisfaction, fatigue, and sleep problems. Structural equation models were used to fit a latent variable model where both expectation variables were used to predict health-related quality of life. Results showed that expectations regarding potential functional improvement resulting from treatments at the Gastein Healing Gallery were associated with improved health-related quality of life. Expectations about general health improvements related to treatment were not associated with health-related quality of life. To facilitate optimal healing, clinicians may decide to emphasize expectations about functional recovery when discussing treatment methods similar to those offered at the Gastein Healing Gallery, and in so doing, health-related quality of life may benefit.

## 1. Introduction

This paper examines Alpine health resources found in Bad Gastein, Austria. Bad Gastein is a small mountain community in the Austrian Alps well known as a spa and ski destination with a longstanding tradition but also as an evidence-based health tourism destination. The Gastein Healing Gallery is located in the small village of Böckstein, near the city of Bad Gastein. Approximately 12,000 patients with inflammatory, rheumatic, and degenerative diseases that result in chronic pain go to the Gastein Healing Gallery each year seeking preventative treatment and rehabilitative services [1]. Patients come to treatment settings and protocols with varying expectations about their chosen course of treatment [2], and this too is the case with the Gastein Healing Gallery. For this reason, we sought to understand how patient expectations were related to the health outcomes of patients attending the Gastein Healing Gallery.

### 1.1. Brief Historical Highlights of Ecomedicine

Speleotherapy can be allocated to the larger group of therapies used in ecomedicine. The origins of ecomedicine might be traced to spa bathing which originated in the Belgian town, Spa, where active thermal springs were discovered in the 14th century [3]. It is thought that the water is supportive for eliciting the relaxation response, thereby activating the vagus nerve and, in turn, encouraging relaxation of the body’s muscles and providing pain relief. Spa bathing has been used for therapeutic and preventive purposes which currently include balneotherapy, spa therapy, and hydrotherapy. Spa therapy includes water treatment but does not limit itself to specifically thermal water treatment [4]. Balneotherapy is defined as the treatment of diseases by bathing in thermal mineral waters in order to treat chronic skin and musculoskeletal conditions [5]. Hydrotherapy refers to specifically using water as therapy in any form [6]. The speleotherapy offered at the Gastein Healing Gallery combines multiple aspects of spa, balneotherapy, and hydrotherapy in unique ways using heat and high humidity but also adds healing elements of mild radon radiation [7,8], a high content of negative air ions [9], and low mountain altitude (1270 m above sea level) [10].

The attractiveness of ecomedicine might be attributed to its non-invasive nature and encouragement of innate self-healing capacities. Spa bathing has been used throughout history, in Ancient Greece and the Roman Empire, during the Renaissance, 19th and 20th centuries, and continues to the present day [3]. Hippocrates proposed that the root of all diseases resulted from an imbalance of bodily fluids, which could only be restored by bathing, perspiration, walking, and massages [3]. The Greeks and Romans built their own thermal baths at mineral and thermal springs to treat afflicted body parts by immersing the entire body in the water and drinking excessive amounts of water [3]. By the turn of the 19th and 20th centuries, thermal water bathing had become more common as a leisure and social activity than a healing process. In recent years, balneotherapy has been widely used as a non-pharmacological intervention for the treatment of rheumatic and other disorders [11]. Balneotherapy has beneficial effects on pain, function, and quality of life [11]. There is also a growing body of knowledge of the biological mechanisms by which balneotherapy and spa treatment alleviate symptoms of several diseases [11,12].

### 1.2. Expectations and Health Behaviors

Research suggests that people who expect medical treatment to provide health benefits are more likely to be motivated to commit to and continue the treatment. Albert Bandura’s long-standing social cognitive theory has highlighted numerous factors influencing the likelihood of executing a health behavior, specifically in health promotion, and one of the key factors is outcome expectations [13]. An example of research testing Bandura’s social cognitive theory is a study of 187 Iranian type 2 diabetes mellitus patients who completed measures of diabetes self-care and outcome expectancies [14]. The results of this study showed a statistically significant positive correlation of moderate magnitude between positive outcome expectancies and improved self-care behaviors.

Another study demonstrating the importance of expectations for health behaviors was conducted in the United Kingdom and examined the effectiveness of an oral health educational program [15]. Participants in this study were divided into treatment and control groups, with the treatment group undergoing four hour-long sessions of oral health education specifically designed to improve self-efficacy and outcome expectancies. Compared with the control group, participants in the intervention who experienced oral health education showed improved oral health behaviors both immediately following the intervention and at a two-month follow-up assessment. The connections between expectations and health behaviors are critical because the promotion of good health behaviors is related to sustainable, long-term health outcomes [16].

### 1.3. Expectations and Health Outcomes

The biomedical model has fallen short in its understanding and treatment of chronic musculoskeletal pain [17]. Hence, attention to psychological factors, including patient and provider expectations, has increased [17]. For example, one study used a five-step approach to shaping clinicians’ expectations toward holistic treatment and is based on the biopsychosocial model of chronic musculoskeletal pain [17]. This approach includes self-reflection, assessment of beliefs, and clinical reasoning and education so that the provider’s and patient’s expectations towards treatment are improved. With increased positive expectations of both patient and provider towards treatment, patients may be able to develop and maintain higher compliance with treatment guidelines, with a greater likelihood of consequent positive clinical outcomes.

Multiple studies have been conducted on the effect of expectations and clinical outcomes in the treatment of musculoskeletal pain. A systematic review examining 52 studies of 28,885 participants showed that individual recovery expectations were associated with the 12-month prognosis of outcomes in patients with non-specific lower back pain [18]. This review found what could be considered moderate-quality evidence that recovery expectations were positively correlated with treatment participation from patients. However, the evidence for an association between recovery expectations and clinical outcomes, such as functional limitations, and pain intensity, were less certain.

Other studies have found similar links between patient expectations and improvement. One randomized clinical trial examined the relationship between expectations and outcomes in 593 patients with low back pain participating in clinical treatment [19]. Results showed that patients with high expectations had a 58 percent chance of reporting a subjective improvement in pain at the fourth visit. The findings also confirmed that baseline psychological profile, pain intensity, and self-rated health did not confound this relationship. Another study of 716 patients with neck pain showed a 47 percent increased probability of improvement after a seven-week follow-up in patients with high expectations for manual therapy [20].

### 1.4. Present Study

Interest in ecomedicine is growing [21] and Alpine health resources are one significant part of this larger movement in medical care. Past research has demonstrated that expectations are important for health promotion behaviors and health outcomes, and understanding patient expectations is a growing and pertinent concern in healthcare [2]. However, few, if any, studies have examined patients’ expectations regarding Alpine health resources such as the Gastein Healing Gallery. Many patients seeking care at the Gastein Healing Gallery are experiencing pain that can progressively erode both overall physical health and create functional limitations. Therefore, the purpose of this study was to understand how treatment expectations regarding overall health and functional limitations were related to health-related quality of life. We examine these two dimensions of health expectations because expectations can be thought of as being similar to attitudes, and decades of attitudinal research have shown the importance of considering both general and specific levels of this construct [22]. To be clear, health expectations are developed in complex and multifaceted ways involving numerous personal, situational, and cultural factors [23]. Nevertheless, the focus here is not on the development of expectations, but on the nature of general health-focused expectations, versus specific functional expectations, and their relation to health-related quality of life. Based on previous research, we hypothesize that individuals who hold more positive expectations toward the treatments offered at the Gastein Healing Gallery will also report better health-related quality of life.

## 2. Materials and Methods

### 2.1. Participants

Participants in this study were 1444 individuals seeking treatment at the Gastein Healing Gallery in Bad Gastein, Austria (see Table 1). The sample consisted of individuals 20–85 years of age (M = 58, Mdn = 58, SD = 11). Women represented 52 percent (N = 751) of the sample and men comprised 48 percent (N = 693) of the sample. Educational status was 25 percent junior high school (N = 359), 48 percent (N = 696) high school, and 27 percent (N = 389) college/university. Occupational status was 39 percent employed (N = 565), 38 percent retired (N = 546), 3 percent homemaker (N = 41), 2 percent disability (N = 30), 1 percent unemployed (N = 17), less than 1 percent students (N = 5), and 17 percent other occupational status (N = 240). Common chronic illnesses of the participants were as follows: ankylosing spondylitis (23%, N = 336), osteoarthritis (14%, N = 195), fibromyalgia (9%, N = 129), and rheumatoid arthritis (5%, N = 77). However, many patients have diagnosed or undiagnosed chronic pain, and a small percentage (<3%) of patients have other diagnosable disorders such as psoriatic arthritis, COPD, asthma, atopic dermatitis, and psoriasis.

### 2.2. Measures

#### 2.2.1. Expectations

Two measures of health expectations were developed. These items were selected from a measure originally developed and validated by Kuhn et al. [24]. Items from this measure that were identified as key indicators of two different dimensions of expectations regarding treatment were selected. Expectations regarding health-related functional capacity were assessed with three items (α = 0.89) reflecting beliefs of improved function resulting from Gastein Healing Galleries treatment. Each item began with the phrase, “If I do rehabilitation with gallery sessions, then…” The three items were: (1) “I will be able to perform strenuous activities again (running fast, lifting heavy things, perform strenuous sports)”, (2) “I will be able to perform moderate activities again (lifting and carrying shopping bags, walking stairs, walking more than 1 km)”, and (3) “I will be able to perform daily chores at home or at my work without difficulties again.” Expectations regarding overall health were assessed with three items (α = 0.93) reflecting aspects of general pain and health. Each item began with the phrase, “I am convinced, that gallery sessions help me…” The three items were: (1) “to reduce my pain”, (2) “to reduce my health troubles”, and (3) “to improve my health status.” All expectation items were responded to on a five-point Likert-type scale of 1 (do not agree) to 5 (completely agree).

#### 2.2.2. Pain

Patients were asked to rate their current pain on a scale from 0 (no pain) to 100 (strongest pain imaginable) [25,26].

#### 2.2.3. Health

Patients were asked to rate their current health on a scale from 0 (worst health) to 100 (best health) [27,28].

#### 2.2.4. Life Satisfaction

A single item measured life satisfaction: “All things considered, how satisfied are you with your life as a whole?” Patients responded on an 11-point scale from 0 (completely dissatisfied) to 10 (completely satisfied) [29].

#### 2.2.5. Depression and Anxiety

Depression and anxiety were measured with the Patient Health Questionnaire (PHQ-9) and the Generalized Anxiety Disorder screener (GAD), respectively. The PHQ-9 is a diagnostic tool for mental health disorders used by healthcare professionals that is quick and easy for patients to complete [30]. The PHQ contains the mood (PHQ-9), anxiety, alcohol, eating, and somatoform modules as covered in the original PRIME-MD. The GAD-7 was subsequently developed as a brief scale for anxiety. The PHQ-9, a tool specific to depression, simply scores each of the 9 DSM-IV criteria based on the mood module from the original PRIME-MD. The GAD-7 scores 7 common anxiety symptoms. Various versions of the PHQ scales are discussed in the instruction manual (https://www.phqscreeners.com/ (accessed on 18 March 2023)). We used the short forms of both screeners with two items measuring depression symptoms and two items measuring anxiety symptoms [31,32,33]. Items assessed how often over a two-week span the patient had been bothered by symptoms of anxiety (e.g., feeling nervous, worrying) and depression (e.g., lack of interest, feeling hopeless). Responses were made on a scale from 0 (not at all) to 3 (nearly every day). Internal consistency was 0.75 and 0.82, respectively, for depression and anxiety scales.

#### 2.2.6. Perceived Stress

Stress was assessed with the four-item version of the Perceived Stress Scale (PSS-4). The PSS-4 assesses the extent to which events in the respondents’ lives are considered stressful [34]. Items address the extent to which respondents feel they cannot control important things in their life, they lack the confidence to handle personal problems, that things are going their way (reverse scored), or that difficulties are becoming insurmountable. The PSS-4 is positively related to depressive, anxious, and physical symptomology, and negatively related to health-related quality of life. The PSS-4 has good validity, but its predictive validity is affected by time and is expected to rapidly fall off as a result of the changing nature of acutely stressful events. Responses were made on a scale of 0 (never) to 4 (very often). The internal consistency of the PSS-4 was 0.73.

#### 2.2.7. Fatigue

The multidimensional fatigue inventory (MFI) is a 20-item instrument intended to assess several key aspects of fatigue including general fatigue, physical fatigue, mental fatigue, reduced motivation, and reduced activity [35]. Respondents indicate the fatigue they have experienced lately on a Likert-type scale ranging from 1 (no, that is not true) to 5 (yes, that is true) indicating how certain statements apply regarding their fatigue. A higher total score is indicative of higher levels of fatigue. The MFI manual recommends the use of the general fatigue subscale as a measure of global fatigue, and that subscale is included in this study. The internal consistency of the MFI was 0.85.

#### 2.2.8. Sleep Problems

Sleep problems were assessed with three items of the Sleep Condition Indicator. This screening tool was developed to evaluate insomnia disorder [36]. We used the two items from the brief version of the scale [37]. For these two items, patients are asked to respond to the prompt, “Thinking about a typical night in the last month…” Items included: (1) How many nights a week do you have a problem with your sleep? (response options: 4 (0–1 nights) to 0 (5–7 nights)). (2) How often has poor sleep troubled you in general? (response options: 4 (not at all) to 0 (very much)). We included a third item to assess the longer-term duration of the problems. This third item asked how long the patient had a problem with their sleep (response options: 4 (I don’t have a problem/<1 month) to 0 (>1 year)). The item scores are added, and a higher score means better sleep. The internal consistency of the sleep problems scale was 0.84.

#### 2.2.9. Demographics

Age was measured in years. Sex was measured as male (coded “1”) and female (coded “0”). Education was measured in categories of 1 (secondary school), 2 (junior high school), 3 (high school), 4 (college), and 5 (university).

### 2.3. Procedure

We used LimeSurvey (Version 3, Limesurvey.org) to conduct an anonymous online survey with patients regularly attending the Gastein Healing Gallery. Invitations were sent to 6465 patients by email to fill out the survey. Patients gave informed consent on their first visit to the Gallery to receive emails for marketing purposes and online surveys. The purpose of the study and the voluntary nature of participant involvement were described at the beginning of the survey. Because this is not an intervention study and data collection and analysis were anonymous, neither an ethical evaluation of the study nor reporting of the study to the Austrian Federal Office for Safety in Healthcare was required (European Legislation Identifier (ELI): https://www.ris.bka.gv.at/eli/bgbl/II/2022/374/20221007 (accessed on 18 March 2023)). This project was conducted in accordance with the Declaration of Helsinki.

### 2.4. Analyses

We used structural equation modeling (Amos 29) to examine the relationships between expectations and health outcomes. Two latent predictors were created to represent expectations for health and expectations for functional limitations. A single latent variable was designed to comprehensively capture variation in patients’ overall health-related quality of life. This variable was comprised of measures of pain, mental and physical health, life satisfaction, fatigue, and sleep problems. Age, sex, and education were used as socio-demographic control variables. The model was evaluated using several model fit indices including chi-square, comparative fit index (CFI), root mean square error of approximation (RMSEA), and standardized root mean square residual (SRMR), and path coefficients are provided in standardized units. Bollen–Stine adjusted fit indices were used because multivariate kurtosis was present in the model. There was no missing data. Statistical significance was set at *p* < 0.05.

## 3. Results

Table 2 provides the means, standard deviations, and Pearson correlations for all study variables used in the factor and structural models. Before testing the structural model, we examined the factor structure of our expectations measure. We examined both one-factor and two-factor models, however, the one-factor model fit the data significantly worse (Δχ^2^ = 239.46, *p* < 0.001) compared with the two-factor model, suggesting that the distinction between expectations about functional limitations due to health and overall health is an important one. In the two-factor model, the fit of the model was good: χ^2^ = 29.26; CFI = 1.0; SRMR = 0.04; RMSEA = 0.04, 95% C.I. 0.03–0.06. All factor loadings were statistically significant and ranged from 0.83–0.95 for general health expectations and 0.73–0.93 for functional limitations expectations. The correlation between the two factors was 0.60.

**Figure 1:**
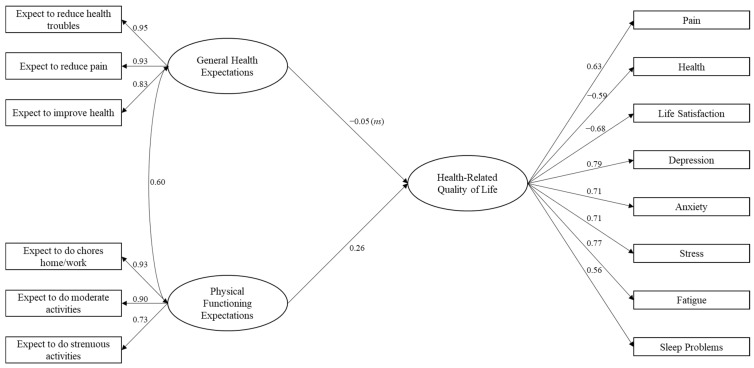
Latent structural model of the associations between general health expectations and physical functioning expectations and health-related quality of life. Note: latent health expectations for improved physical functioning predict health-related quality of life (β = 0.26, *p* < 0.001). Latent general health expectations do not predict health-related quality of life (β = −0.05, *p* = 0.146). Age (β = −0.14, *p* < 0.001), sex (β = −0.12, *p* < 0.001), and education (β = −0.17, *p* < 0.001) were all significant predictors and were controlled but are not shown in the figure. All coefficients are standardized and are statistically significant at *p* < 0.001 unless otherwise noted (ns = nonsignificant).

When testing the structural model (see Figure 1) we observed a good-fitting model (χ2 = 148.42; CFI = 1.00; SRMR = 0.04; RMSEA = 0.02). All measured indicators significantly loaded (*p* < 0.001) on their respective latent factors. Loadings for expectations of health ranged from 0.83–0.95, and loadings for expectations of functional limitations ranged from 0.73–0.93. Loadings on the health-related quality of life variable ranged from 0.56–0.79. Age (β = −0.14, *p* < 0.001), sex (β = −0.12, *p* < 0.001), and education (β = −0.17, *p* < 0.001) were all significant predictors of health-related quality of life. Expectations for overall health were not a significant predictor (β = −0.05, *p* = 0.146), but expectations for functional limitations (β = 0.26, *p* < 0.001) were a significant predictor of health-related quality of life.

## 4. Discussion

Our structural model showed that expectations about the effects of the Gastein Healing Gallery on improved functional limitations were related to improved overall health-related quality of life. Expectations about improved overall health were not related to health-related quality of life. This is an interesting outcome. Perhaps expectations for improved health are not sufficient motivators for engagement in health behaviors and treatment that bring about the same gains to health-related quality of life, as compared with expectations for functional improvement. In short, patients may need to expect more than to just feel a better sense of health. They may need to expect that engagement in Gastein Healing Galleries treatments, and health-promoting behavior in general, will bring back a more complete integration into their broader lives [38]. In sum, our findings suggest that patients who expect to become more functional and capable show a better health-related quality of life.

Our findings are consistent with several studies showing that expectations positively correlated with health-related quality of life outcomes in patients with chronic pain [14,15,16,19,20,39]. Published work has shown that expectations predict higher self-reported improvement in patients with low back pain who were participating in clinical treatment [19]. Similarly, increased improvement occurred in patients with neck pain who had positive expectations for therapy [20]. It is interesting to note that our assessments were focused on correlations between expectations with health-related quality of life, and not with clinical outcomes as in other studies. Given that self-reported health-related quality of life is often one of the most robust predictors of beneficial physical health outcomes [38], our findings suggest that enhancing expectations for functional improvement may be an important therapeutic strategy.

However, not all studies have found a positive association between expectations and clinical outcomes. One study of 28,885 patients with non-specific lower back pain showed that recovery expectations were positively linked to treatment participation, but less so with clinical outcomes such as functional limitations and pain intensity [16], suggesting that expectations may have differential associations with health behaviors versus health outcomes. As such, our study proposed a novel distinction between expectations about engaging in ecomedical treatment and its benefits for reduced functional limitations compared with the impact on overall health. Our finding, that only patients who expected to become more functional and capable of healthy behavior showed improved health-related quality of life, emphasizes the point that it is critical to understand the matrix of distinct expectations and their connections to relevant behavioral, clinical, and health-related quality of life outcomes.

Our findings are consistent with Bandura’s social cognitive theory, which emphasizes the role of people’s attitudes and beliefs in models of health [13]. Bandura’s theory maintains that reinforcing people for healthy behavior is more effective than punishing them for lack thereof and highlights the importance of social support systems in changing the self-regulatory habits of individuals. As such, one of the key factors influencing the likelihood of health behavior execution is expectations, and many studies have empirically confirmed this relation. For example, outcome expectancies have been shown to be positively correlated with self-care behaviors during treatment in people with diabetes [14], and self-efficacy and outcome expectancies have been related to improved oral-health behaviors in female students in the United Kingdom [15]. These findings coincide with our results indicating that expectations about functional limitations are a strong indicator of greater overall health-related quality of life, most likely through increased execution of health behavior. Future work to investigate this potential mediating link will be important.

There is growing evidence for a comprehensive biopsychosocial view of chronic pain symptoms and syndromes. As such, factors including patients’ attitudes and beliefs, clinical reasoning, and expectations, as supported by clinicians, are especially important to consider alongside a treatment’s inherent biological action [17]. We see the prominence of psychological factors in mind-body treatment options such as mindfulness, tai-chi, and yoga interventions. Indeed, a recent review article on integrative medicine interventions for treating episodic migraine concluded that mindfulness meditation can be a powerful tool to improve quality of life, decrease affective response to pain, and decrease headache frequency in patients who experience medication withdrawal [40]. These approaches often focus on changing perceptions and expectations regarding one’s experience of health and well-being.

Another important facet of the discussion regarding patient expectations is the well-known placebo effect, in which patients’ expectations, in addition to learning histories, have a positive effect on treatment outcomes [41]. The placebo effect has been well-documented to be therapeutic in various randomized clinical trials with chronic pain conditions such as migraine, neuropsychological conditions such as age-related cognitive decline, insomnia, epilepsy, and depression [42]. Despite prevalent support for the therapeutic placebo effect, there have also been contradictory findings and a lack of evidence in many conditions where mind-body medicine is a popular treatment option [42]. To offer additional evidence that may help to address this discrepancy, the present study has shown that in Alpine treatment of chronic pain syndromes, psychosocial factors such as patients’ expectations likely play an important, positive role in health-related quality of life, alongside the well-known beneficial biological mechanisms of the therapy.

As with all studies of this type, there are some limitations to take into consideration. First, our study used online self-report questionnaires, and the inherent limitations and biases of such measurement are likely present herein. For example, we cannot verify if the patient or someone else completed the survey. Second, the sample consisted of only patients who chose to attend an alpine health resource center. As such, there may be a selection bias that results in a favorable predisposition towards this type of treatment which may enhance positive expectations for treatment outcomes. Further, we do not know if surveys were completed before or after visits to the Gastein Healing Gallery. Third, our population was diverse in age, sex, educational status, and health issues, however, it was limited in racial, ethnic, cultural, and national diversity. Fourth, our sample is limited to those patients with internet connections. Last, our study design is cross-sectional, thus, causality between expectations and the overall health-related outcome cannot be determined.

## 5. Conclusions

The purpose of our study was to examine how expectations were related to health-related quality of life in patients with chronic pain syndromes attending the Gastein Healing Gallery in Böckstein near Bad Gastein, Austria. Our results suggested that expectations about reduced functional limitations were a strong predictor of overall health-related quality of life, while expectations regarding the effects of Gastein Healing Gallery on overall health were not related to health-related quality of life. Given the growing interest in ecomedicine due to the low cost, less invasive nature, and promising results, future studies should continue to evaluate the effects of expectations and other psychosocial factors on treatment outcomes in chronic pain patients, as well as in patients with other conditions where complementary and integrative medicine is popular. Qualitative study designs might offer deep insights into patient expectation development and the substance of expectations. Studies examining the biopsychosocial mechanisms by which expectations confer their influence on health-related quality of life would be of good utility. Furthermore, experimental designs should also be implemented to establish causality between expectations about functional limitations and healthier behaviors that eventually result in overall health-related quality of life in patients with chronic pain syndromes utilizing Alpine health resources.

## Figures and Tables

**Table 1:** Participant demographic characteristics.

Variables	N (%)/M ± SD
Age	58 ± 11
Sex	
- Females	751 (52)
- Males	693 (48)
Education	
- Junior high school	359 (25)
- High school	696 (48)
- College/university	389 (27)
Occupational status	
- Employed	565 (39)
- Retired	546 (38)
- Homemaker	41 (3)
- Disability	30 (2)
- Unemployed	17 (1)
- Student	5 (<1)
- Other occupational status	240 (17)
Chronic illnesses	
- Ankylosing spondylitis	336 (23)
- Osteoarthritis	195 (14)
- Fibromyalgia	129 (9)
- Rheumatoid arthritis	77 (5)
- Other diagnosable disorders or undiagnosed chronic pain	707 (49)

**Table 2:** Means, standard deviations, and Pearson correlations for all study variables.

Variable	M	SD	1		2		3		4		5		6		7		8	
1. Age	58.16	10.84	--															
2. Sex (1 = male, 0 = female)	1.48	0.50	0.15	***	--													
3. Education	2.59	1.40	0.04		−0.03		--											
4. Expect to improve health	1.55	0.91	0.05		0.01		0.04		--									
5. Expect to reduce pain	1.63	0.98	0.07	**	−0.02		0.10	***	0.76	***	--							
6. Expect to reduce health troubles	1.63	0.95	0.08	**	−0.02		0.08	**	0.78	***	0.88	***	--					
7. Expect to do strenuous activities	2.60	1.20	0.11	***	−0.04		0.01		0.42	***	0.40	***	0.41	***	--			
8. Expect to do moderate activities	2.01	1.18	0.06	*	−0.05		0.04		0.50	***	0.50	***	0.50	***	0.66	***	--	
9. Expect to do chores at home/work	2.08	1.15	0.03		−0.06	*	0.02		0.52	***	0.52	***	0.52	***	0.68	***	0.84	***
10. Pain	4.17	2.28	−0.06	*	−0.09	***	−0.19	***	0.05	*	−0.04		0.02		0.15	***	0.10	***
11. Health	66.10	20.03	−0.01		0.07	**	0.14	***	−0.11	***	−0.04		−0.08	**	−0.24	***	−0.16	***
12. Life satisfaction	7.32	2.01	0.12	***	0.06	*	0.08	**	−0.11	***	−0.09	***	−0.11	***	−0.16	***	−0.15	***
13. Depression	0.79	0.73	−0.16	***	−0.10	***	−0.18	***	0.09	***	0.02		0.06	*	0.13	***	0.10	***
14. Anxiety	0.60	0.67	−0.12	***	−0.14	***	−0.05	*	0.05		0.00		0.04		0.10	***	0.10	***
15. Stress	1.42	0.77	−0.11	***	−0.10	***	−0.12	***	0.06	*	0.04		0.05	*	0.09	***	0.10	***
16. Fatigue	2.02	1.03	−0.15	***	−0.14	***	−0.10	***	0.10	***	0.05		0.09	***	0.20	***	0.15	***
17. Sleep problems	1.89	1.35	−0.06	*	−0.14	***	−0.07	**	0.05	*	−0.01		0.05		0.12	***	0.10	***
**Variable**	**M**	**SD**	**9**		**10**		**11**		**12**		**13**		**14**		**15**		**16**	
9. Motivated to do chores at home/work	2.08	1.15	--															
10. Pain	4.17	2.28	0.13	***	--													
11. Health	66.10	20.03	−0.20	***	−0.53	***	--											
12. Life satisfaction	7.32	2.01	−0.19	***	−0.36	***	0.46	***	--									
13. Depression	0.79	0.73	0.15	***	0.52	***	−0.43	***	−0.52	***	--							
14. Anxiety	0.60	0.67	0.12	***	0.38	***	−0.32	***	−0.45	***	0.64	***	--					
15. Stress	1.42	0.77	0.13	***	0.38	***	−0.34	***	−0.57	***	0.55	***	0.58	***	--			
16. Fatigue	2.02	1.03	0.19	***	0.50	***	−0.47	***	−0.50	***	0.61	***	0.51	***	0.54	***	--	
17. Sleep problems	1.89	1.35	0.13	***	0.41	***	−0.37	***	−0.35	***	0.39	***	0.38	***	0.39	***	0.48	***

* *p* < 0.05, ** *p* < 0.01, *** *p* < 0.001.

## Data Availability

The data presented in this study are available on request from the corresponding author. The data are not publicly available due to the need to protect the confidentiality of patient medical records and self-report data.
